# Pharmacokinetic Profile of Inhaled Submicron Particle Paclitaxel (NanoPac^®^) in a Rodent Model

**DOI:** 10.1089/jamp.2018.1467

**Published:** 2019-03-29

**Authors:** James Verco, William Johnston, Michael Baltezor, Philip J. Kuehl, Andrew Gigliotti, Steven A. Belinsky, Anita Lopez, Ronald Wolff, Lauren Hylle, Gere diZerega

**Affiliations:** ^1^ US Biotest, Inc., San Luis Obispo, California.; ^2^ CritiTech, Lawrence, Kansas.; ^3^ Lovelace Biomedical, Albuquerque, New Mexico; ^4^ RK Wolff–Safety Consulting, Fort Myers, Florida.; ^5^ NanOlogy, LLC, Fort Worth, Texas.

**Keywords:** chemotherapy, inhalation, nab-paclitaxel, NanoPac, NSCLC, paclitaxel, pharmacokinetics, rat, rodent

## Abstract

***Background:*** Inhaled chemotherapeutics may enhance pulmonary drug exposure to malignant lesions in the lung without substantially contributing to systemic toxicities. The pharmacokinetic profile of inhaled submicron particle paclitaxel (NanoPac^®^) in healthy rodent plasma and lung tissue is evaluated here to determine administration proof-of-principle.

***Methods:*** Healthy male Sprague Dawley rats received paclitaxel in one of three arms: intravenous nab-paclitaxel at 2.9 mg/kg (IVnP), inhaled NanoPac low dose (IHNP-LD) at 0.38 mg/kg, or inhaled NanoPac high dose (IHNP-HD) at 1.18 mg/kg. Plasma and lung tissue paclitaxel concentrations were determined using ultraperformance liquid chromatography tandem mass spectrometry from animals sacrificed at 10 time points ranging up to 2 weeks after administration. Peak concentration (C_max_), apparent residence half-life (T_1/2_), exposure (AUC_(last)_), and dose-normalized exposure (AUC_D(last)_) were determined. Pulmonary histopathology was performed on rats sacrificed at the 336-hour time point.

***Results:*** Paclitaxel was detectable and quantifiable in the rat lung for both inhaled NanoPac arms sampled at the final necropsy, 336 hours postadministration. Substantial paclitaxel deposition and retention resulted in an order of magnitude increase in dose-normalized pulmonary exposure over IVnP. Inhaled NanoPac arms had an order of magnitude lower plasma C_max_ than IVnP, but followed a similar plasma T_1/2_ clearance (quantifiable only to 72 hours postadministration). Pulmonary histopathology found all treated animals indistinguishable from treatment-naive rats.

***Conclusion:*** In the rodent model, inhaled NanoPac demonstrated substantial deposition and retention of paclitaxel in sampled lung tissue. Further research to determine NanoPac's toxicity profile and potential efficacy as lung cancer therapy is underway.

## Introduction

The American Cancer Society estimates that in 2018 the United States will diagnose 243,030 new cases of lung cancer, and 154,050 deaths. Lung cancer will be responsible for 25% of all cancer-related deaths, accounting for more than breast, prostate, and colon cancer combined.^(1)^ Surgical treatment for localized disease provides the best prognosis in early stage disease; however, early diagnosis of lung cancer is challenging, with 57% of patients exhibiting distant metastasis upon symptomatic presentation. For patients diagnosed with metastatic disease, it is estimated that only 4.5% will survive an additional 5 years.^(1–3)^

Surgery for these patients is generally no longer viable, requiring intravenous chemotherapy, immunotherapy, radiotherapy, or a combination thereof to prolong survival, control symptoms, or improve quality of life. Recent advances in targeted- and immunotherapies have substantially increased overall survival for their specific corresponding subpopulations,^(4–6)^ yet the majority of patients with lung cancer will only exhibit a survival benefit of a few months. Therefore, the need remains to provide an additional long-lasting benefit to patients without significantly affecting quality of life.

A review by Nichols et al. evaluated mortality in 100 lung cancer patients, 91 of whom had presented with metastatic disease. The study found that local disease progression as the principle cause of death in 59% of subjects, causative factors included primary tumor burden, pulmonary hemorrhage, pulmonary thromboembolism, and diffuse alveolar damage.^(7)^ Additional therapy administered directly to the primary cancer may, therefore, provide an enhanced survival response even in patients exhibiting extrapulmonary disease. Increasing local drug exposure through conventional intravenous methods widely distributes drug to nontarget organs, leading to severe systemic toxicities and low bioavailability to pulmonary malignancies before clearance by metabolism and excretion.^(8)^

Inhaled chemotherapy provides an alternate route of administration to overcome limitations associated with intravenous methods; substantial doses can be delivered directly to the lung while avoiding additive toxic exposure to nontarget vital organs.^(8–10)^ Historically, achieving increased pulmonary exposure through inhalation is limited by poor retention of drug within the lung due to clearance mechanisms such as diffusion across the alveolar–capillary barrier, the mucociliary escalator removing material to the gastrointestinal tract, phagocytosis by alveolar macrophages and dendritic cells, and lymphatic drainage.^(11–13)^

To remain clinically feasible, inhaled chemotherapeutics would preferentially have the ability to evade or minimize pulmonary clearance mechanisms, while continuously releasing drug to maintain constant exposure to malignant lesions.^(11)^ Inhaled cisplatin,^(14,15)^ doxorubicin,^(16,17)^ carboplatin,^(18)^ gemcitabine,^(19)^ and 9-nitro-camptothecin^(19)^ have demonstrated proof-of-principle in clinical safety studies, yet despite promising results, have not progressed to complete efficacy trials.

Paclitaxel, an FDA-approved intravenous cytotoxic taxane that induces mitotic arrest through stabilization of microtubules in the G2/M cell cycle phase,^(20,21)^ has yet to be clinically evaluated for efficacy as an inhaled chemotherapeutic for the treatment of NSCLC. Owing to paclitaxel's poor solubility, commercially available formulations for systemic administration pose unique difficulties for deposition and retention in the lung following inhalation. The first approved paclitaxel formulation Taxol^®^ requires 50% Cremaphor EL as a solvent, and is known to elicit toxic biological effects.^(22,23)^ The subsequently approved albumin-bound paclitaxel (Abraxane^®(24)^) has been studied preclinically for inhaled administration, which demonstrated remarkably efficient systemic bioavailability.^(25)^

Varying coated formulations of paclitaxel have exhibited beneficial pharmacokinetic profiles^(11,26–29)^ and antitumor efficacy in both rodent^(11,28–32)^ and canine models.^(33,34)^ Of note, not only did inhaled paclitaxel significantly inhibit tumor growth as measured by lung weight,^(30–32)^ but also significantly inhibited pulmonary metastasis from intravenously administered kidney cancer cells (Renca cell line).^31^

### NanoPac^®^

NanoPac^®^ is paclitaxel processed utilizing precipitation with compressed antisolvents, into uncoated submicron crystals sized between 600 and 800 nm^35,36^ that allow for suspension reconstitution in physiological saline containing 0.1% polysorbate 80 (PS80). Designed large enough to avoid clearance by systemic circulation, but with high internal surface area for drug release characteristics of smaller particles, local administration of NanoPac directly at malignant sites provides a depot effect releasing paclitaxel into surrounding fluids and tissues at constant saturation levels.^36^

Although *in vitro* dissolution methods have been marginally useful for evaluating the release of paclitaxel from NanoPac, unpublished *in vivo* trials and a phase 1 intraperitoneal clinical study have demonstrated the extended release profile. Twenty-one patients with peritoneal malignancies received intraperitoneal (IP) doses of 50–275 mg/m^2^, in up to six cycles (every 28 days); the peritoneal fluid paclitaxel concentrations were 450–2900 times greater than systemic plasma, resulting in fewer toxic-related events in comparison with intravenous trials.^36^

With its slow depot release properties, aerosolized NanoPac may have the potential to provide a meaningful survival benefit for patients with lung cancer. Following nebulization and deposition within the alveolar sacs, rapid diffusion of the saline would theoretically leave NanoPac particles behind for constant release of bioavailable paclitaxel directly to pulmonary surfactant and malignant cells. In this study we explore the feasibility and pharmacokinetics of nebulized NanoPac for inhalation therapy in a rodent model.

## Materials and Methods

All animal procedures were conducted under protocols approved by the Institutional Animal Care and Use Committee (IACUC) at Lovelace Biomedical (Albuquerque, NM), which is accredited by the Association for Assessments and Accreditation of Laboratory Animal Care International.

### Animals

Male Sprague Dawley rats (6–8 weeks old) were obtained from Charles River Laboratories (Kingston, NY). The rats were quarantined for 14 days, after which the animals were weighed and randomized for study assignment. Animals were identified by tail marking and cage card. Water, lighting, humidity, and temperature control were maintained and monitored. Rats were fed a standard rodent diet *ad libitum* during nonexposure hours.

### Chemicals and reagents

Paclitaxel USP reference standard (USP Catalog No.: 1491332) and Paclitaxel-13C6 (TRC Catalog No.: P132504) were used as reference and internal standards within the LCMS assay. USP reference standard was used within the HPLC assay as the reference standard.

NanoPac dry powder and reconstitution solution were provided to Lovelace Biomedical by NanOlogy, LLC (Lawrence, KS), and were prepared as follows. In brief, 5.0 mL of 1% Polysorbate 80 (PS80) was added to the dry NanoPac powder vial, which was shaken vigorously and inverted to ensure wetting of all particles. Immediately after shaking, 0.9% sodium chloride solution (Hospira, IL) was added to the NanoPac vial and shaken for at least 1 minute to ensure proper dispersion, 46 mL for the 6.0 mg/mL suspension and 10.3 mL for 20.0 mg/mL suspension. Resultant formulations were left undisturbed for at least 5 minutes to reduce any air/foam in the vial before placing it in a jet nebulizer for aerosolization. The composition of the varying NanoPac concentrations only differs in final concentration of PS80: 0.1% and 0.33% for the 6.0 and 20.0 mg/mL suspensions, respectively.

Lovelace Biomedical obtained nab-paclitaxel, Abraxane (Celgene Corporation, NJ), from a clinical pharmacy and drug was reconstituted to 5.0 mg/mL with saline on the day of dosing, stored and administered per manufacturer's instructions.

### Paclitaxel quantification verification from spiked glass fiber filter (GF/A)

Aerosol filter paclitaxel analysis was conducted, non-GLP, with an Agilent 1100 HPLC-ultraviolet (HPLC-UV) (Santa Clara, CA) fitted with a Phenomenex Hypersil ODS (C18) (Torrance, CA). The column temperature was set at 25°C with detection at 227 nm. The flow rate was 1.0 mL/min and the injection volume was 5 μL. Quantification was performed within validated Empower 3 software with a seven-point standard curve between 5 and 300 μg/mL prepared with Paclitaxel USP reference standard. Before utilization, the assay was characterized for linearity, reproducibility, accuracy, and precision.

In addition, a filter (GF/A, Whatman) extraction method was developed and characterized for recovery (target 85–115% recovery) over the range of paclitaxel on filters (90–785 μm). A liquid extraction was performed in 7 mL glass vials with 4 mL of methanol and acetic acid diluent (200:1). The samples were rotated, vortexed, and centrifuged at 13,000 revolutions per minute (rpm), and transferred to autosampler vials for HPLC analysis.

Sample analysis was performed with linearity standards and quality control checks within every run. The filter data were processed within Empower with validated custom fields to determine the amount of paclitaxel on filter and the corresponding paclitaxel aerosol concentration.

### Paclitaxel quantification verification from biological samples

Sample (rat plasma and lung tissue) paclitaxel analysis was conducted through a non-GLP ultraperformance liquid chromatography tandem mass spectrometry (UPLC-MS/MS), with an ABSciex API 4000 triple quadrupole mass spectrometer (Framingham, MA) coupled to an Acquity H-Class UPLC (Waters, Milford, MA) with ABSciex Analyst version 1.6 software. Sample extracts were resolved on an Acquity UPLC BEH C(18) column.

Quantification was performed with paclitaxel reference standard (USP) and internal standards (Paclitaxel-^13^C_6_) in matrix (Rat plasma K_2_EDTA and naive lung tissue). Quality control checks were prepared in matrix over the range of each method. Plasma extraction was performed through protein precipitation with acetonitrile (100 μL plasma with 300 μL of acetonitrile). Lung tissue samples included a homogenization in 1 × phosphate-buffered saline (PBS), 1 g lung tissue with 4 mL PBS before the same protein precipitation. Samples were prepared and assayed with quality controls (QCs run in triplicate) at 3, 80, and 800 ng/mL to assess the accuracy and precision of paclitaxel. Curve fitting was performed using linear regression with 1/*x^2^* weighting requiring a produced correlation coefficient ≥0.98.

Within bioanalytical runs, standard criteria were applied for standard inclusion (±15%) and QC criteria (±15%) before run acceptance and release of the sample data. All data below the range of the assay were reported as BQL (below quantification level).

### Treatment protocol and sampling

**FIG. 1.**
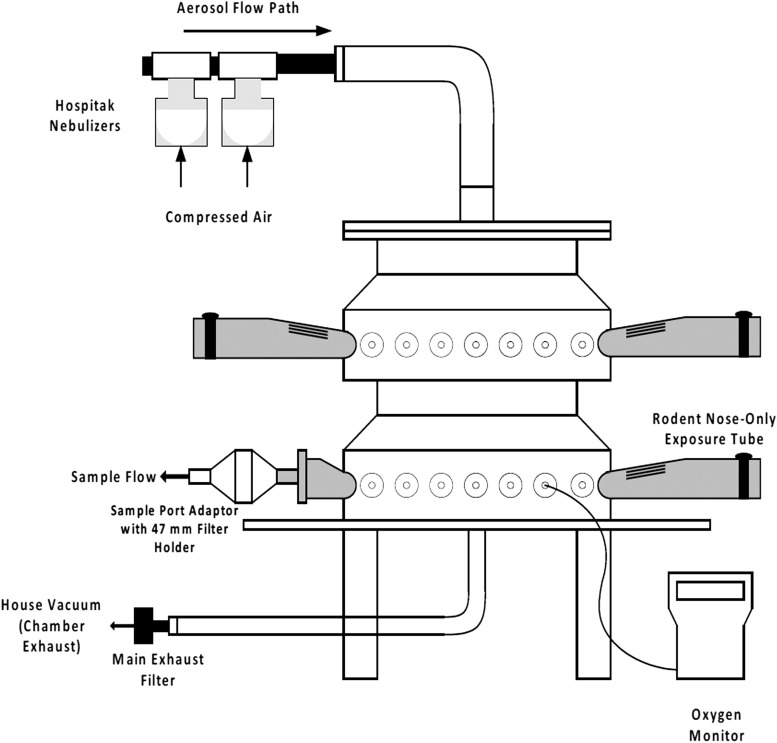
Schematic of the rodent exposure system for nebulized NanoPac aerosol.

Ninety animals were randomized into three treatment groups (*n* = 30). The first group of animals were to receive a single intravenous tail vein injection of nab-paclitaxel (IVnP) as a positive treatment control to assess inhaled NanoPac feasibility, and was dosed to a maximum allowable 5.0 mg/kg or corresponding dose associated with the maximum allowable injection volume of 250 μL as set by IACUC. NanoPac suspension formulations of 6.0 mg/mL and 20.0 mg/mL were aerosolized on a single occasion for the inhaled NanoPac low-dose (IHNP-LD) and inhaled NanoPac high-dose (IHNP-HD) arms, respectively. Both formulations were aerosolized separately and were directed through a delivery line into a 32-port nose-only exposure chamber for 65 minutes (Fig. 1).

NanoPac aerosols were generated with a set of two Up-Mist (Hospitak, ConvaTec, McAllen, TX) compressed air jet nebulizers at a nebulizer pressure of 20 psi (used for up to 40 [±1] minutes), then replaced with a second set of two Up-Mist nebulizers for the remaining exposure duration for a total exposure time of 65 minutes. Oxygen and temperature were monitored and recorded throughout each inhalation exposure.

Three animals (*n* = 3) from each arm were sacrificed at 0.5 (±10 minutes), 6 (±10 minutes), 12 (±10 minutes), 24 (±30 minutes), 48 (±30 minutes), 72 (±30 minutes), 120 (±30 minutes), 168 (±30 minutes), 240 (±30 minutes), and 336 (±30 minutes) hours postexposure. Blood samples for plasma bioanalytical analysis were collected via cardiac puncture into K_2_EDTA tubes. Whole lung weight was measured, lung lobes were separated, individually weighed and snap frozen in liquid nitrogen, and stored at −70°C to −90°C. Right lung lobes were designated for bioanalytical analyses and left lung lobes for histopathological review. External surfaces of the body, orifices, and the contents of the cranial, thoracic, and abdominal cavities were examined.

### Particle and aerosol characteristic determination

Particle size distribution of aerosols was measured from rodent breathing zone of the nose-only exposure chamber by a Mercer-style, seven-stage cascade impactor (lntox Products, Inc., NM). Cascade impactor samples were collected at a flow rate of 2.0 ± 0.1 L/min. The particle size distributions were determined in terms of mass median aerodynamic diameter (MMAD) and geometric standard deviation (GSD). Aerosolized NanoPac concentration monitoring was conducted by analyzing aerosols on preweighed GF/A 47-mm filters sampled every 10 minutes at a flow rate of 1.0 ± 0.5 L/minute. Filters were weighed to determine the total aerosol concentration in the exposure system, then extracted and analyzed by HPLC methods already mentioned to quantify paclitaxel collected on each filter.

### Inhaled dose determination

Deposited dose could not be determined *in vivo* due to active clearance in the lung and administration occurring for 65 minutes. Deposited pulmonary dose was calculated using Equation 1, using the average paclitaxel aerosol concentration measurements, body weights, and administration windows. Varying MMAD sizes results in different fractions of total aerosol to be deposited within the lung; FDA assumes 10% deposition fraction for rodents inhaling particles with MMAD 1–5 μm.^37,38^ Previous analysis with Hospitak nebulizers assumed nebulization droplets to fall within this range for the Up-Mist nebulizer.

Equation 1: Inhaled deposited dose calculation.^39^

\begin{align*}
DD \; \left( { \mu g / kg } \right) = { \frac { AC \, \left( { \mu g / L } \right) \, \times \,RMV \; \left( { L / min } \right) \, \times \,DF \, \times \,T \, \left( { { \rm { min } } . } \right) }  { BW \; \left( { kg } \right) } } ,
\end{align*}

where DD is deposited dose, BW is body weight, RMV is respiratory minute volume = 0.608 × BW^0.852^, AC is aerosol concentration, and DF is deposition fraction.

### Pharmacokinetics

Plasma samples were thawed immediately before analysis and were assayed through UPLC-MS methods already mentioned to quantify paclitaxel concentration at the specified necropsy time points. Right lung lobes were thawed, homogenized with PBS at a ratio of 4:1 (water–lung tissue), and underwent a similar protein precipitation with acetonitrile before UPLC-MS analysis. Quantification was conducted with a matrix-based calibration curve, with a lower level of quantification determined from the biological sample verification methods already mentioned. Noncompartmental analysis (Phoenix WinNonlin 6.2 [Certara, Princeton, NJ]) was conducted on average data at each time point from the plasma and lung tissue concentrations. Noncompartmental analysis was performed to calculate C_max_, T_1/2_, AUC_(last)_, and AUC_D(last)_.

### Histopathology

Histopathologic examination was performed on left lung lobes from animals in all treatment arms (*n* = 3 per arm) necropsied at the final time point (336 hours), alongside three untreated controls. Tissues were processed routinely, paraffin embedded, sectioned at 4 μm, mounted, and stained with hematoxylin and eosin (H&E) for microscopic examination. Findings were graded subjectively and semiquantitatively by a single pathologist experienced in toxicologic pathology. The Provantis™ (Instem LSS Ltd., Staffordshire, England) computer software/database was used for histopathology data acquisition, reporting, and analysis.

## Results

### Clinical observations, survival, and body weights

All animals survived to their designated necropsy time points and were euthanized within the intended windows. No abnormal clinical observations, signs of distress, or labored breathing were noted throughout the study duration. Every animal had an increased body weight at necropsy in comparison with administration except for a single animal in the IHNP-LD group necropsied 6 hours after administration, which experienced a 9% loss in body weight.

### Paclitaxel quantification verification from spiked glass fiber filter (GF/A)

The HPLC assay, before utilization, was shown to be linear between 5 and 300 μg/mL (correlation coefficient of linearity curve [*R*^2^] was 0.9989) and reproducible (% RSD of 0.3% between repeated injections). The filter spike recovery showed recovery of no more than 3.6% from target.

### Paclitaxel quantification verification from biological samples

The bioanalytical methods (plasma and lung tissue) were characterized before use for accuracy, recovery, precision, and linear range. The assay was linear between 1 and 1000 ng/mL for plasma, and 10–10,000 ng/g for lung tissue, with correlation coefficients of ∼0.99. The accuracy and precision of the assay met standard ±15% range for small molecule criteria. Recovery was assessed in plasma between 3 and 800 ng/mL and in lung tissue between 10 and 7500 ng/g, all of which met standard ±15% range for small molecule criteria.

### Particle and aerosol characteristics and dose determination

**FIG. 2.**
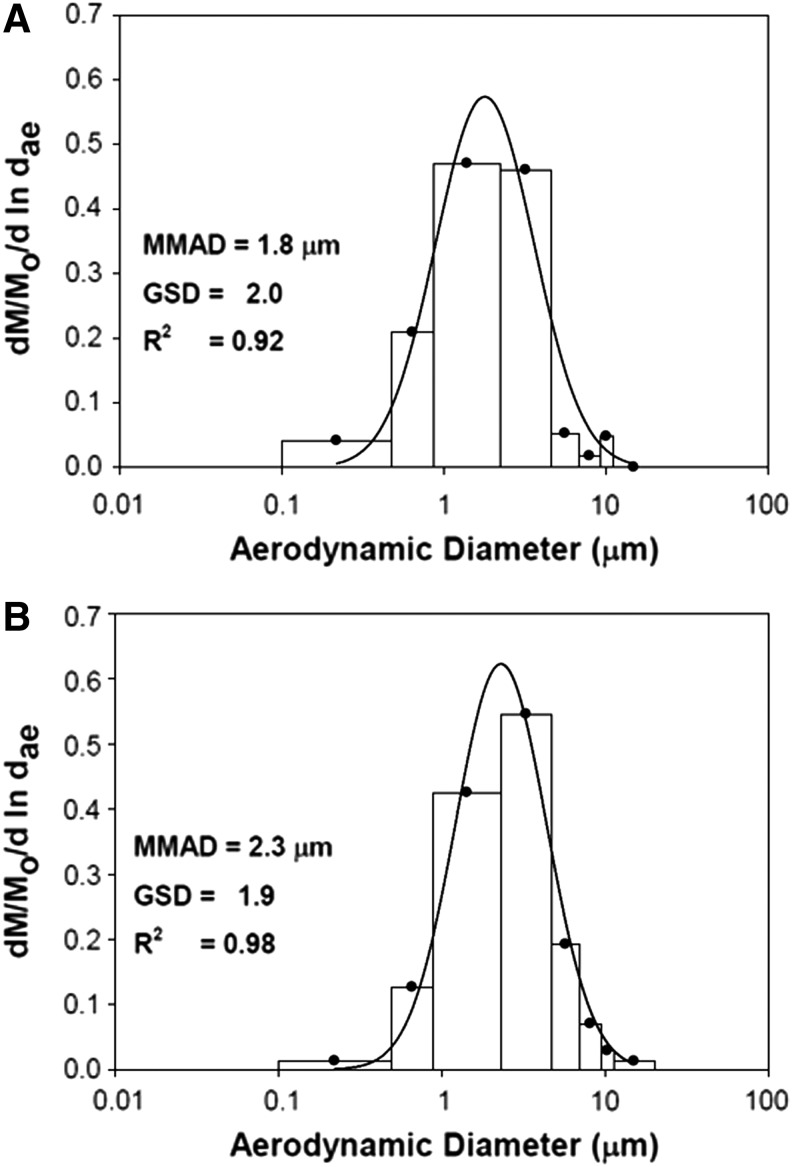
Mass median aerodynamic size distribution of nebulized **(A)** 6.0 mg/mL NanoPac suspension and **(B)** 20.0 mg/mL NanoPac suspension. NanoPac aerosols generated by two Up-Mist compressed air jet nebulizers produced aerosols with MMAD (GSD) of 1.8 (2.0) μm and 2.3 (1.9) μm as measured with the Mercer-style cascade impactor for the 6.0 mg/mL and 20.0 mg/mL suspensions, respectively. MMAD, mass median aerodynamic diameter; GSD, geometric standard deviation; *R*^2^ = coefficient of correlation.

NanoPac aerosols generated by two Up-Mist compressed air jet nebulizers running simultaneously, producing aerosols with MMAD (GSD) of 1.8 (2.0) μm and 2.3 (1.9) μm (Fig. 2) for the aerosolized 6.0 and 20.0 mg/mL suspension formulations, respectively. Filters from both aerosol exposure groups were analyzed for paclitaxel accumulation and evaluated into average paclitaxel aerosol concentration (summarized in Table 1). Average paclitaxel aerosol concentrations (standard deviation) were 85.64 (8.76) μg/L and 262.27 (31.45) μg/L for the 6.0 and 20.0 mg/mL suspension formulations, respectively. Estimated doses of 0.38 and 1.18 mg/kg were calculated for the IHNP-LD and IHNP-HD arms, respectively. Owing to increasing body weights and maximum allowable injection volume of 250 μL, the IVnP arm received an average dose of 2.9 mg/kg.

**Table 1.** Inhaled Aerosolized NanoPac Exposure Filter Analysis

	*Filter ID No.*	*Total aerosol concentration (mg/L)*	*Paclitaxel aerosol concentration (μg/L)*
IHND-LD	FS-1-L	0.247	80.05
	FS-2-L	0.242	81.79
	FS-3-L	0.252	87.09
	FS-4-L	0.296	104.38
	FS-5-L	0.247	78.47
	FS-6-L	0.249	82.50
	FS-7-L	0.244	85.19
	Average	0.25	85.64
	SD	0.02	8.76
	% RSD	7.43	10.23
IHND-HD	FS-1-H	0.383	212.53
	FS-2-H	0.412	239.28
	FS-3-H	0.494	291.44
	FS-4-H	0.516	296.56
	FS-5-H	0.456	254.67
	FS-6-H	0.501	289.50
	FS-7-H	0.431	251.88
	Average	0.46	262.27
	SD	0.05	31.45
	% RSD	10.95	11.99

IHNP-LD, inhaled NanoPac low-dose arm; IHNP-HD, inhaled NanoPac high-dose arm; SD, standard deviation; % RSD, percentage relative standard deviation.

### Pharmacokinetics of inhaled NanoPac

**FIG. 3.**
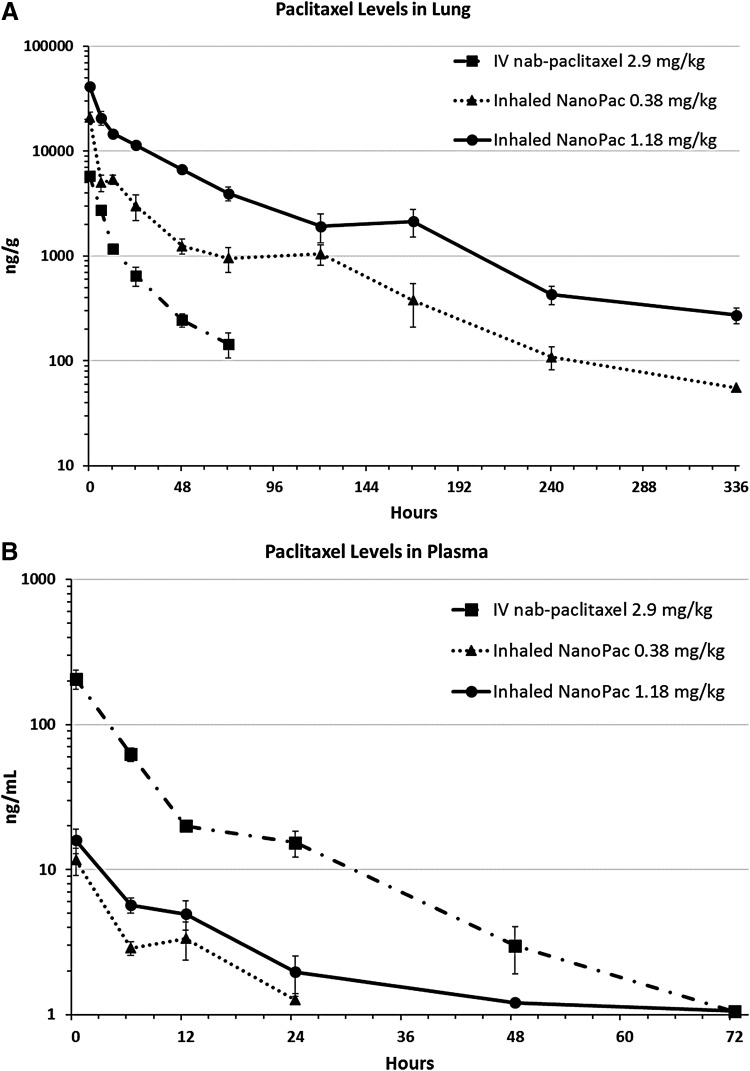
Postadministration paclitaxel concentration levels **(A)** in the lung tissue and **(B)** in the plasma. Male Sprague Dawley rats (6–8 weeks old) were administered paclitaxel on a single occasion in one of three treatment arms (*n* = 30 each): inhaled NanoPac in a nose-only exposure at a low dose of 0.38 mg/kg or a high dose of 1.18 mg/kg, or intravenous nab-paclitaxel administered through tail vein injection at 2.9 mg/kg. Three animals from each arm were sacrificed at 0.5, 6, 12, 24, 48, 72, 120, 168, 240, and 336 hours postexposure for lung tissue and plasma collections. Lung tissue **(A)** and plasma **(B)** were assayed through ultraperformance liquid chromatography tandem mass spectrometry to quantify paclitaxel concentration as a function of time with a lower level of quantification of 50 ng/g and 1 ng/mL, respectively (mean ±1SEM).

Tables 2 and 3 depict analyzed paclitaxel concentrations from plasma and homogenized right lung tissue, respectively. Table 4 summarizes the calculated pharmacokinetic values for both the plasma and lung tissue. The paclitaxel concentration–time curves throughout the study for the lung tissue and plasma are visualized in Figure 3A and B, respectively.

**Table 2.** Paclitaxel Quantification in Rodent Plasma

*Time point (hour)*	*IVnP*			*IHNP-LD*			*IHNP-HD*		
	*Concentration (ng/mL)*	*Average (ng/mL)*	*Standard deviation*	*Concentration (ng/mL)*	*Average (ng/mL)*	*Standard deviation*	*Concentration (ng/mL)*	*Average (ng/mL)*	*Standard deviation*
0.5	153	206	44.1	15.6	11.6	3.5	10.8	15.9	4.3
	205			12.1			21.3		
	261			7.09			15.6		
6	70.5	62.2	9.2	3.44	2.87	0.4	6.56	5.69	1.0
	66.7			2.37			4.35		
	49.3			2.81			6.15		
12	18.9	20.0	0.9	5.29	3.35	1.4	7.14	4.95	1.6
	20			2.08			3.47		
	21.1			2.67			4.23		
24	9.46	15.3	4.4	BQL	1.26	0.1	1.47	1.96	0.8
	16.3			1.16			3.11		
	20.1			1.36			1.31		
48	5.08	2.98	1.5	BQL	BQL	N/A	1.21	1.21	N/A
	1.56			BQL			BQL		
	2.3			BQL			BQL		
72	BQL	1.05	N/A	BQL	BQL	N/A	BQL	1.06	N/A
	1.05			BQL			1.06		
	BQL			BQL			BQL		
120	BQL	BQL	N/A	BQL	BQL	N/A	BQL	BQL	N/A
	BQL			BQL			BQL		
	BQL			BQL			BQL		
168	BQL	BQL	N/A	BQL	BQL	N/A	BQL	BQL	N/A
	BQL			BQL			BQL		
	BQL			BQL			BQL		
240	BQL	BQL	N/A	BQL	BQL	N/A	BQL	BQL	N/A
	BQL			BQL			BQL		
	BQL			BQL			BQL		
336	BQL	BQL	N/A	BQL	BQL	N/A	BQL	BQL	N/A
	BQL			BQL			BQL		
	BQL			BQL			BQL		

IVnP, intravenous nab-paclitaxel arm (2.9 mg/kg); IHNP-LD, inhaled NanoPac-low dose (0.38 mg/kg); IHNP-HD, inhaled NanoPac-high dose (1.18 mg/kg); BQL, below quantifiable level; N/A, not assessable.

**Table 3.** Paclitaxel Quantification in the Right Rodent Lung

*Time point (hour)*	*IVnP*			*IHNP-LD*			*IHNP-HD*		
	*Concentration (ng/g)*	*Average (ng/g)*	*Standard deviation*	*Concentration (ng/g)*	*Average (ng/g)*	*Standard deviation*	*Concentration (ng/g)*	*Average (ng/g)*	*Standard deviation*
0.5	5850	5800	430.1	19,450	21,000	3503.1	40,400	41,600	1557.8
	5250			17,700			43,800		
	6300			25,850			40,600		
6	2665	2730	106.4	6700	4990	1219.1	15,500	20,800	4499.6
	2880			3945			20,400		
	2645			4325			26,500		
12	1045	1170	113.7	6200	5368	764.1	17,050	14,700	1661.8
	1145			5550			13,500		
	1320			4355			13,550		
24	386	647	188.6	2325	3008	1170.0	10,300	11,433	838.0
	825			2045			11,700		
	730			4655			12,300		
48	307	244	48.1	850	1247	288.9	6000	6700	535.4
	190			1530			7300		
	237			1360			6800		
72	101	145	54.0	950	950	355.2	4375	3953	863.5
	221			1385			4735		
	113			515			2750		
120	BQL	BQL	N/A	1500	1045	327.1	1570	1923	846.1
	BQL			890			1110		
	BQL			745			3090		
168	BQL	BQL	N/A	309	377	236.1	3395	2143	889.4
	BQL			695			1410		
	BQL			129			1625		
240	BQL	BQL	N/A	58	109	38.4	271	430	122.8
	BQL			151			448		
	BQL			117			570		
336	BQL	BQL	N/A	BQL	55.5	N/A	233	272	67.5
	BQL			55.5			367		
	BQL			BQL			216		

IVnP, intravenous nab-paclitaxel arm (2.9 mg/kg); IHNP-LD, inhaled NanoPac-low dose (0.38 mg/kg); IHNP-HD, inhaled NanoPac-high dose (1.18 mg/kg); BQL, below quantifiable level; N/A, not assessable.

**Table 4.** Paclitaxel Pharmacokinetic Profiles

*Group*	*Average paclitaxel aerosol concentration μg/L (std dev)*	*Dose mg/kg (std dev)*	*Plasma*				*Lung tissue*			
			*C_max_ ng/mL (std dev)*	*T_1/2_ hours*	AUC_(last)_ hr^*^ng/mL	AUC_D(last)_ hr^*^ng^*^mg/mL^*^kg	*C_max_ ng/g (std dev)*	*T_1/2_ hours*	AUC_(last)_ hr^*^ng/g	AUC_D(last)_ hr^*^ng^*^mg/g^*^kg
IVnP	N/A	2.9 (0.16)	206 (44.1)	8.7	1,517	528	5,800 (430.1)	19.9	62,870	23,112
IHNP-LD	85.64 (8.76)	0.38 (0.003)	11.6 (3.5)	7.9	101	264	21,000 (3503.1)	56.3	342,877	914,095
IHNP-HD	262.27 (31.45)	1.18 (0.01)	15.9 (4.3)	8.6	228	193	41,600 (1557.8)	56.0	1,155,662	997,985

C_max_, maximum concentration; T_1/2_, apparent residence half-life; AUC_(last)_, exposure (area under the curve); AUC_D(last)_, dose-normalized exposure; std dev, standard deviation.

The initial paclitaxel exposure to the right lung lobes of the rats was higher in the IHNP-LD and IHNP-HD arms than in IVnP, with C_max_ values 3.5- and 7-fold greater, respectively. Although paclitaxel quantification was not performed on the left lung lobes, they were assumed to have similar concentration profiles as the right lobes.^40^ After deposition, inhaled NanoPac appeared to clear from the lung at a slower rate than that of IVnP, a roughly threefold increase in T_1/2_ in both arms. IVnP paclitaxel levels were only quantifiable to the 72-hour time point, whereas both inhaled NanoPac arms were quantifiable to study completion (336-hour time point).

The paclitaxel deposition and retention resulted in a pulmonary AUC_(last)_ roughly 5.5- and 18-times greater in the IHNP-LD and IHNP-HD arms than in the IVnP arm, respectively. When dose normalized, IHNP-LD and IHNP-HD arms exhibited 39- and 43-fold greater paclitaxel exposure per drug unit dose than the IVnP arm.

The plasma paclitaxel concentration was higher in the IVnP arm than in the IHNP-LD and IHNP-HD arms, with C_max_ values in the plasma roughly 17.75- and 13-fold greater, respectively. All arms exhibited similar clearance from the plasma, all T_1/2_ values being within a range of 7.9–8.7 hours. Furthermore, IVnP and IHNP-HD were only quantifiable to the 72-hour time point, and IHNP-LD only to 24-hour time point. These plasma paclitaxel levels resulted in an AUC_(last)_ exposure 15- and 6.5-fold lower in the IHNP-LD and IHNP-HD than in IVnP, respectively. Dose-normalized plasma exposures were also greater in the IVnP arm by roughly 2- and 2.75-times than in the IHNP-LD and IHNP-HD arms, respectively.

### Histopathology

**FIG. 4.**
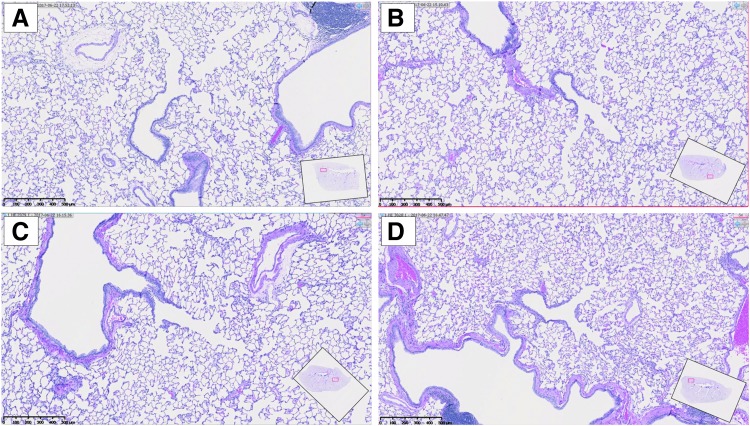
Histology slides from **(A)** an untreated rat, **(B)** a rat treated with intravenous nab-paclitaxel at 2.9 mg/kg, **(C)** a rat treated with inhaled NanoPac at 0.38 mg/kg, and **(D)** a rat treated with inhaled NanoPac at 1.18 mg/kg (bar = 500 μm). Tissues from fixed left lung lobes of rats sacrificed at the 336-hour time point were processed routinely, paraffin embedded, sectioned at 4 μm, mounted, and stained with hematoxylin and eosin for microscopic examination.

No abnormalities were noted within the trachea or left lung lobes of the animals sacrificed 336 hours postadministration in any arm. When compared with untreated control tissue samples, treated tissues were microscopically indistinguishable (Fig. 4). Although not be construed as equivalent to a thorough histologic evaluation, the results indicate that 2 weeks postsingle administration of NanoPac at 1.18 mg/kg, substantial pathology does not occur within the trachea or lungs.

## Discussion

Inhalation of aerosolized NanoPac was successful in this study via compressed air jet nebulization. Particle characteristic analysis found both NanoPac suspensions nebulized with MMADs ∼2 μm, falling within the 1–3 μm range required for efficient distribution and deposition throughout the lung when translating into the clinic.^41–47^ Paclitaxel quantification on the right lung lobes found retention of drug beyond 14 days, whereas systemic plasma concentrations fell BQL 3 days postadministration. This extended retention of paclitaxel at malignant sites may allow for increased efficacy when combined with conventional therapies to treat diseases such as NSCLC, without substantially contributing to systemic toxicities.

Although the total paclitaxel concentration analyzed within the right lung lobes is considerable, the amount of bioavailable paclitaxel is still to be determined as release from NanoPac is gated by saturation levels in the surrounding environment. Although residual NanoPac crystals may remain within the lung up to 2 weeks after inhalation, histologic evaluation of the left lung lobes from rats sacrificed at 336 hours postadministration revealed treatment arms indistinguishable from untreated controls. Multiple administration pharmacology and toxicology studies are underway to further research inhaled NanoPac's potential as lung cancer therapy.

## Ethical Adherences

This study complied with all applicable sections of the Final Rules of the Animal Welfare Act regulations (9 CFR Parts 1, 2, and 3), as well as the Guide for the Care and Use of Laboratory Animals (2011). Lovelace Biomedical is fully accredited by the Association for Assessment and Accreditation of Laboratory Animal Care.

## Notes

Reviewed by: Remi Rosiere
